# Diffusion of innovation, pre‐registration/licence student nurses' use of social media: A reflexive thematic analysis

**DOI:** 10.1111/jan.16405

**Published:** 2024-08-20

**Authors:** Xabi Cathala, Calvin Moorley

**Affiliations:** ^1^ School of Health & Psychological Sciences City St George's, University of London London UK; ^2^ School of Nursing and Midwifery Institute of Health & Social Care, London South Bank University London UK

**Keywords:** diffusion, professionalism; survey, social identity, social media

## Abstract

**Aim:**

To identify and report the use of social media among pre‐registration (pre‐licence) student nurses.

**Methods:**

A social survey was conducted in 2019 to explore student nurses' views of social media usage. Diffusion of innovation theory and social identity theory were used as the theoretical framework. A reflexive thematic analysis was undertaken of responses to an open‐ended question.

**Results:**

351 responses were analysed. Four themes emerged: Social media as a communication tool that helps to keep in contact and communicate with friends, family, colleagues and peers; Social media and self‐care including a sense of pride, boosting morale and helping to relax; Social media and learning by sharing experiences, chatting, posts and social media and professionalism, participants expressed confusion over the use of social media professionally.

**Conclusions:**

The diffusion of social media among UK student nurses is advancing in different aspects of UK student nurses' lives. However, the diffusion seems to slow down when it comes to the professional system. They express concerns that one social system can negatively impact another and have repercussions on a personal and/or professional level and therefore prevent UK student nurses from developing social media proficiencies. Guidance and support should be offered to UK student nurses to develop their social identity across the different systems. This diffusion can help to educate student nurses and future professionals in a globally connected world.

**Impact:**

Social media features in student nurses' personal and professional lives and presents challenges for social identity which is woven into the personal and professional personas. Nursing faculties should have social media competencies embedded into the curricula to develop and strengthen students' social and professional identities across the different systems.

**Patient or Public Contribution:**

No Patient or Public Contribution.

## 1 INTRODUCTION

Social media are seen as a place for connecting, talking, sharing ideas and chatting. However, it has educational purposes which are often overlooked. Social media is increasingly being used for educational and professional learning. Social media are online platforms driven by users that support the diffusion of content, dialogue and communication and encourage interactions and networking at personal, professional and societal levels (Ohara, 2023). Social media are involved in multiple aspects of our daily lives (Maitri et al., 2023), and the nursing community does not escape the phenomenon (Daigle, 2020; Lefebvre et al., 2020; Zhu et al., 2021) as seen in the guidance offered. The International Council of Nurses (2015) position statement on nurses and social media acknowledges its potential to strengthen the nursing profession. The UK Nursing and Midwifery Council guidance (2018) similarly states if used appropriately several benefits can be realized for nurses. Ryan's (2016) content analysis on international perspectives, though limited to the UK and New Zealand, concludes that these guidelines are essential and continuous updates are crucial for the evolving landscape. Steers and Gallups (2020) believe these guidelines can help nurses maintain the ethical balance of a social media presence.

The younger generation, students from Generation Z (1995–2015) onwards, has only known life with the Internet. Their personal, educational and professional lives are surrounded by social media and technology (Hampton et al., 2020; Mohr & Mohr, 2018; Mude & Undale, 2023).

## 2 BACKGROUND

The population investigated are student nurses attending universities in the United Kingdom (UK). The nursing literature concerning social media is growing. Price et al. (2018) and Greenhow and Galvin (2020) reported that teaching and incorporating social media into learning activities benefit students. With younger generations accessing higher education, these generational needs must be investigated, understood and considered by nurses, nurse educators, higher education institutions, stakeholders and associations to adapt education and support student nurses to use social media. Our search identified one scoping review that focused on pre‐registration nursing education and the diffusion of social media. In this review of 27 papers, Cathala and Moorley (2023) found that nursing students adopted social media in their learning much faster than their educators. Picton (2023) doctoral thesis, examined undergraduate diagnostic radiology students' use of social media and reported that personal usage confidence is high, while professional usage confidence is low, and the intervening gap needs to be addressed if the benefits of using social media to augment learning during studies are to be realized. Based on Cathala and Moorley (2023) and Picton (2023) work, it can be argued that this disparity creates a gap between student learning needs and university strategy curriculum delivery.

Due to the lack of empirical evidence and robust methodologies, further research is recommended (Cathala, Ocho, Watts, & Moorley, 2021; Lefebvre et al., 2020; Scott & Goode, 2020; Terzi̇ et al., 2019).

Two theories were used to guide the exploration of student nurses' experience using social media. First, the theoretical framework of the research project is the diffusion of innovation theory developed by Everett Rogers in 1962 (Rogers, 2003). This theory was applied in different fields to investigate the diffusion of new technology, ideas and inventions. Rogers defined and developed the diffusion process incorporating four elements: the innovation; the innovation's communication channels; time; and the social system (Rogers, 2003). The second theory, the social identity theory (SIT), was developed by Tajfel, a British psychologist, in 1979. Tajfel and Turner (2004) proposed three consecutive processes within social identity theory supporting the development of social identity: Social categorization, Social identification and Social comparison. Social identity theory was used in this study to place students' experiences into context in the diffusion of social media innovation.

## 3 AIM

To identify and report the use of social media among UK student nurses and align its use to the social media diffusion timeline by using Rogers' (2003) diffusion of innovation theory as a theoretical framework to answer the research question: How does social media diffuse among pre‐registration (pre‐licence) UK student nurses?

## 4 METHODS

A social survey was developed based on existing literature and was conducted to explore UK and Caribbean student nurses' views of social media usage and the quantitative results have previously been published (Cathala, Ocho, Watts, & Moorley, 2021). This paper reports on a reflexive thematic analysis of the qualitative data elicited from an open‐ended question in the survey and reports only on the UK respondents and was analysed following the Overviewing Conceptual and Design Thinking for Thematic Analysis Research developed by Braun and Clarke (2022). Reflexive thematic analysis engages procedures and practices that embody the values of qualitative approaches. Adopting this stance in the research helped to centre our researcher subjectivity (an area we are familiar with and partial towards) and acknowledge our biases (we use social media as educators in the classroom) while at the same time allowing the discovery of new knowledge (opening our minds to new possibilities and staying focussed on the research question and remaining close to the participants' narrative). The use of two researchers enabled us to sense‐check ideas and explore multiple assumptions or interpretations of the data.

Sociological studies commonly use social surveys to gather large amounts of social data which can include qualitative responses (Payne & Payne, 2011). A qualitative approach was used to analyse the responses to the single question: “*Please tell us about your experience of using social media or why you do not use it?”*. Qualitative analysis attempts to preserve data gathered in its textual form and uses it to generate analytical categories and theoretical explanations (Bailey, 2008). This study upheld the tenets of research on transferability, credibility, trustworthiness and dependability (Lincoln & Guba, 1985).

### 4.1 Participants

The sample is a subset of a larger international sample that included student nurses from the UK, Trinidad and Tobago and Jamaica using purposeful sampling. The data collection section described how the survey was distributed to student nurses across the three countries. The survey was promoted and distributed via participating universities' virtual learning environments and on social media. A set of inclusion and exclusion criteria were used (Table 1). The responses of the Caribbean student nurses were previously analysed and published (Cathala et al., 2022). This study focuses on the qualitative data from a sub‐set (*n* = 351) of UK student nurses who responded to an open‐ended question in the survey: “*Please tell us about your experience of using social media or why you do not use it?”*. The total UK sample comprised 832 student nurses.

**TABLE 1:** Inclusion/exclusion criteria.

Inclusion criteria	Exclusion criteria
Any student nurse enrolled on a pre‐registration nursing programme	Anyone who is not a student nurse or enrolled on a pre‐registration nursing programme
Student nurses on any year of the programme	Any student nurse who is not from either of the participating countries
Student Nurses from Jamaica and Trinidad & Tobago	

### 4.2 Data collection

To ensure an audit trail (credibility, transferability, dependability and confirmability) (Moorley & Cathala, 2019) and good research practice (including ethical approval, instrument pilot testing, data analysis and reflexivity), a flowchart outlining and guiding the research process for data collection was designed. Each participating country received a researcher pack (Table 2). A survey link and a Participant Information Sheet were shared on each participating university's virtual learning environment to advertise the study. Responses were tracked during data collection, and weekly advertisements were posted on universities' virtual learning environments for the first week. The data collection was scheduled for 3 months but extended to 6 (March to September 2019), due to recruitment challenges in the Caribbean.

**TABLE 2:** Research pack.

1.	Participant information sheet
2.	Survey link
3.	Invitation post to use on the virtual learning environment
4.	A thank you email for all participants.
5.	The recruitment process, including the responses monitoring strategy.

For survey design and development, including piloting and data cleaning, see Cathala, Ocho, Moorley, and Watts (2021); Cathala, Ocho, Watts, and Moorley (2021); Cathala et al. (2022). These papers' findings demonstrate that social media are embedded in student nurses' learning and professional development with some specificity related to their country, generation and year of the programme. UK student nurse participants (73.4%, *n* = 618) reported that social media could impact their career less compared to participants from Jamaica and Trinidad and Tobago (80.45%, *n* = 184). Student nurses in Caribbean countries also used and integrated social media in their learning more than in the UK (H(2) = 97.7, *p* < .001; H(2) = 24.4, *p* < .001). Therefore, the qualitative responses can help to identify and report the diffusion of social media among pre‐registration (pre‐licence) UK student nurses' experience using social media.

### 4.3 Ethical considerations

Ethical approval was granted from London South Bank University (Ethical approval number: ETH1819‐0049). We ensured no coercion by reminding students at every stage that participation was voluntary and did not impact their studies. Anonymity was reiterated, and data were protected using a university password‐locked server ensuring General Data Protection Regulations and local ethical compliance. The first question of the survey was a consent question, and any participants who did not consent were excluded. Participants were informed that once the survey was submitted, they could not be withdrawn.

### 4.4 Data analysis

Reflective thematic analysis using Braun and Clarke (2006, 2012, 2022) analytic framework was performed (Table 3). The authors started the analysis by familiarizing themselves with the data by reading the participants' responses. Following the familiarization, the authors started to individually code the data and then generate initial themes. After generating the initial themes, they met to discuss, review and develop their coding and themes. They resolved any differences by discussing and returning to the narratives to ensure congruence. This initiated and supported a reflective analysis. The authors returned to the data coding with the research question in mind and ensured that the themes were related to the coding and dataset. The authors engaged further in the reflective analysis being aware of the impact of their previous knowledge and research findings on the subject. Then, the authors met to review and develop themes from their further coding and initial themes. They refined, defined and named the themes (Table 4). At the final stage of analysis, the researchers selected compelling extracts with analytic commentary. Data analysis was undertaken using NVivo® 12 software version 12.7 to support coding and theme development.

**TABLE 3:** Braun and Clarke (2006, 2012, 2022), thematic analysis framework.

Phase	Description
1. Familiarizing yourself with your data:	The researchers uploaded data onto NVivo software. Data were cleaned and read actively, analytically and critically, noting down initial ideas.
2. Coding the data:	Researchers undertook a coding of the data across the entire data set, collating data relevant to each code.
3. Generating initial themes:	Researchers collated codes into themes, gathering all data relevant to each theme on NVivo
4. Reviewing and developing themes:	Researchers checked if the themes work in relation to the coded extracts and the entire data set.
5. Refining, defining and naming themes:	Researchers undertook ongoing analysis to refine each theme, and the overall story the analysis tells, generating clear definitions and names for each theme.
6. Producing the report:	At the final stage of analysis researchers selected compelling extracts. Final analysis of selected extracts, relating back of the analysis to the research question and literature, producing a scholarly report of the analysis.

**TABLE 4:** Theme definitions.

Theme	Definition
Social Media and communication	Any communication using social media
Social Media and selfcare	Social media use for personal health and wellbeing purposes
Social Media and Learning	Any shared learning experience using social media
SoMe and professionalism	Any shared professionalism experience using social media

### 4.5 Validity and reliability/rigour

**FIGURE 1:**
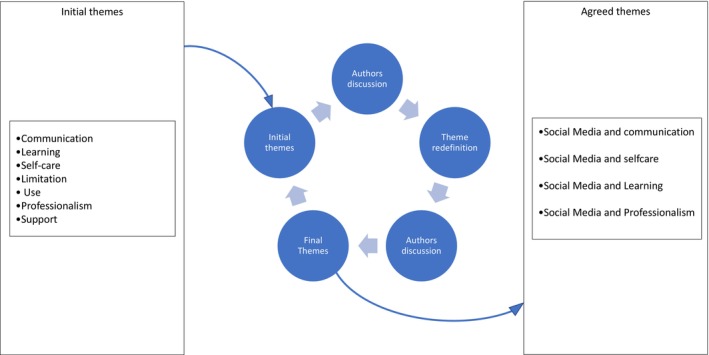
Theme development process.

Initial data‐cleaning for the survey involved removing participants who did not consent and those with missing data. For this qualitative analysis, participants with responses less than 20 words were also removed, to ensure the inclusion of richer, more in‐depth responses. In total, 351 responses were analysed. Two authors analysed the data separately at first and then met and discussed the findings. Using NVivo for analysis, a first read was made for data familiarization (phase 1), reading each qualitative statement to create the database also aided familiarization. The second read was initial coding (phase 2) here the researchers read with a purpose that was focused on the research question and how the participants' narratives answered the questions. Then, the topic summaries referred to as “Themes” were developed which was done by highlighting recurrent words, phrases and statements that shared the same meaning (phase 3). Verbatim extracts were used to support researchers' reflexive thematic analysis. In phases 4, 5 and 6 the authors reviewed, defined, named and produced the analytical report. Main themes were identified (Table 4). Final themes and subthemes were established (Figure 1). Memos were made to aid in moving back and forth through the data, which added clarity to the development of the themes, and verbatim quotes were used for the final report, these demonstrate the trustworthiness, credibility and dependability of the study.

## 5 RESULTS

UK participants (*n* = 351) were 60.1% (*n* = 211) from England, 35.3% (*n* = 124) from Scotland, 4.3% (*n* = 15) from Wales and 0.3% (*n* = 1) from Northern Ireland. The sample represents 4 different generations: 0.3% (*n* = 1) from Boomer (1955–1965), 6.6% (*n* = 23) from Generation X (1966–1976), 45.3% (*n* = 159) from Generation Y (1977–1994) and 47.9% (*n* = 168) from generation Z (1995–2015). Four themes emerged: social media and Communication, social media and self‐care, social media and Learning and social media and professionalism.

### 5.1 Social media and communication

Communication emerged strongly as a theme with 102 responses relating to communication. Social media are viewed as an important part of daily life as seen in the participant's 12 comments.

Social media is an important aspect of daily living in this century so, I use it for socialising and connect with friends. (Participant 12)

Participants' responses below also show that social media helps to keep in contact and communicate with friends, family, colleagues and peers.

Social media has many benefits as it allows you to reconnect or/and find other people. You are then able to share information with them especially close families & friends. (Participant 35).

Social media for me is an important part of my life, as it helps me to stay connected and it also makes me more aware of the current situations, both professional and personal. (Participant 135).

More than a communication tool some participants described social media as a power, for example:

Social media have Definitely given us the power to connect people living on opposite sides of the world. It allows us to talk ‘face to face’ but always behind a screen. (Participant 8)

A significant characteristic of social media to these participants is that it is free to use:

WhatsApp offers free local and international unlimited calls to other WhatsApp users so long as you are connected to a WiFi (Participant 91).

I use it for news mostly now as you are able to access the newspapers I want to read free of charge. I like it to keep up to date with friends (Participant 304).

Most participants reported positive experiences in using social media, as illustrated by these responses.

Always has been a positive experience and in particular twitter as this is a perfect place to be able to connect with other student nurses (Participant 206).

I've had a positive experience of using social media as a nursing student, through being able to connect with others who are able to help me and broaden my knowledge. (Participant 220).

Social media were identified as having downsides without clearly articulating them.

Social media makes it easier to know what is happening currently but also has a downside to it. (Participant 11).

### 5.2 Social media and self‐care

From participants' responses, 24 comments were linked to self‐care, including a sense of feeling proud and boosting morale that the use of social media can provide:

I use it to post my achievements to boost self‐esteem and photos of my children which make me proud. (Participant 1).

A good way of communicating with colleagues and sharing ideas. Helps boost morale with a network of friends. (Participant 171).

Other participants stated that social media helps to decompress, escape stress and anxiety and feel happy, as demonstrated in the comments below:

My social media use has been overwhelmingly positive. I'd normally use it to decompress after a long day, e.g., a quick scroll through Instagram etc. (Participant 32).

I use other platforms – though not as much as Twitter – including Instagram which I only use for things that make me happy (like puppies) and it always cheers me up. Sounds silly but you have to take a smile when you get one. (Participant 153).

Participants expressed the benefit of social media in increasing the feeling of belonging and not being alone, as seen in the following extract:

Social media is quite beneficial whilst being a student Nurse. For example sharing memes and experiences helps one to feel like they are not alone. This course is quite taxing so its nice to not feel alone (Participant 47).

It can make connections that make the student nurses' journey feel less lonely, for example:

I feel part of an online community in certain student nurse groups on twitter and I like to refer to it when I feel stressed or to share experiences. (Participant 89).

During placements with long 12 hour shifts it can be very lonely for student nurses if we are the only student on the ward and due to the shifts it can be difficult to physically spend time with friends and family. It can be very difficult and lonely when coping with the stresses of new environments and learning new things, social media allows me to reach out to friends and other student nurses for support and to share experiences through private messages. (Participant 280).

Not only experiencing personal and individual self‐care but some participants also looked after friends and social media users who might need support.

If I notice a friend who often uses social media, isn't using it, I know that something might be wrong so it enables me to check up on them to make sure they are ok. Sometimes they may just be busy, but sometimes they are withdrawing. (Participant 68).

### 5.3 Social media and learning

The third theme identified was learning and included 85 responses. A large part of those comments described learning between social media users from sharing experiences, chatting and posts. Social media was considered to help to build professional networks:

Professionally I learn from other people posts about medical conditions, but I've also learnt a lot about how people present themself on social media and how they portray in person (Participant 1).

Social media is good to use where you can get to meet and socialise with people and learn from other people experiences (Participant 9).

It is considered useful for academic success in terms of study groups and knowledge acquisition:

It allows me to understand the thinking of others. But it gives me a voice to say what I need to, to the right people. Plus, it helps to gather as much information that I need to learn and aid others in finding out issues that they need to help them with their study. (Participant 131).

I have taken part in many tweet chats which have improved my knowledge and informed my future practice. It's an amazing tool if used correctly. (Participant 137).

Some participants accessed specific pages and groups where they can find information and support for their learning.

On Instagram, I follow some pages of health professionals, a couple of nurses, doctors, Physician's Associate and a few medical pages. They post experiences whilst maintaining confidentiality. They also post about conditions, medications and many other healthcare topics/issues (Participant 10).

Others expressed how social media helps with learning:

Yet now I'm using it to help further my understanding and gain advice from other NQN's and nursing students through a group called student nurse and beyond which is set up as a formal advice group and is moderates by NQN's so that all posts are relevant and don't breach NMC guidelines. I find this helpful as you are able to ask questions and receive responses from students from all over the UK, so the breadth of knowledge is incredible. (Participant 32).

Other students referred to how social media can help to build communities of practice:

I'm now on the WeStudentNurse team helping other students and helping others interact better on Twitter (Participant 77).

Twitter has proven a really positive way of connecting and learning, be it following patient groups that are concerned with a specific condition, following individuals in healthcare who are championing particular causes, e.g. Rob Hackett and his theatre cap challenge, connecting with peers, e.g. The student nurse project, connecting with employers following and interacting with health boards accounts, learning, e.g. following notable lecturers and other organisations that share interesting posts, e.g. Accadoodle. (Participant 94).

Participants reported different positions from the university regarding the use of social media for learning. Some universities were supportive and developed the use of social media in learning as per comments below:

I love that our uni introduced us to digital professionalism from the start and I am enjoying all the networking and learning through my professional Twitter account. I have learnt so much from it and would highly recommend it (Participant 39).

Some students described how social media was embedded in the curriculum, which they considered supports professionalism:

I have a Twitter that I use professionally only as I have no interest in Twitter but my lecturers encouraged us to have some sort of online presence which is professional and for classroom activities. (Participant 201).

Told by the university when we started not to talk about anything to do with nursing on social media. Feel that this has changed massively in the past year and is being encouraged as something that can promote learning opportunities and network with other HCPs. Social media is a great recourse when used appropriately but little guidance has led to some people writing information that could lead to a breach of confidentiality (Participant 210).

However, other participants described resistance or fear from universities in the use of social media in learning:

There is a fear surrounding social media and students using it. Universities scaremonger students that they should not use it and fail to mention the benefits. In addition, the majority of leadership staff are ignorant about the benefits of social media use. (Participant 136).

I really enjoy using social media but I know that my university frowns upon using it even within the social media guidance provided by the NMC and RCN (Participant 250).

Some students expressed a positive impact on their learning and wished they had accessed social media earlier:

I feel Twitter has been a beneficial extension to my learning at university. I have benefited from accessing articles and publications from experts in their fields of practice. I wished I had accessed it earlier in my studies. (Participant 121).

However, other participants reported barriers and limitations to the use of social media for learning:

I feel I waste a lot of time on social media but since joining Twitter I have found I am learning much more about my chosen career as I use this for the professional use. (Participant 132).

I find twitter to be a very good place to grasp some nursing knowledge and create a network but it can also quickly become overwhelming as there is a lot of information coming from everyone, as a result I do not spend a lot of time on it and/or make the most of it. (Participant 42).

### 5.4 Social media and professionalism

The final theme was professionalism, which included 48 responses. Participants expressed confusion over the use of social media, resulting in not using it as a professional, as illustrated by the following extracts:

I do not use social media for professional reason because I don't understand where to draw the line yet as a professional. (Participant 12).

We have a lot of warnings over bad use of social media and put off it in the first year. I shut down all my accounts because of this. There seem to be mixed ideas now over if we should or shouldn't use social media. (Participant 24).

I think the guidelines are not quite clear enough so I don't use social media as much to talk about personal nursing experiences. Rather be safe than sorry. (Participant 225).

Participant 174 stated that social media rules were applied differently depending on whether you were a student nurse or a qualified nurse.

I think it can be really positive but I think some of the rules are incredibly strict. It makes students feel like they cannot share feelings or experiences and that their opinions/time at university is invalid. It should be remembered that we are also students. Many qualified nurses have more relaxed social media rules than students and I think rules should be relaxed (obviously within reason). For example people knowing which trust you work for, really isn't that outrageous and I think sometimes the rules have definitely been taken far too far and have actually impacted us negatively when social media should be used positively. (Participant 174).

Participant 111 agreed with the content monitoring and report of misconduct.

However, I think they should always be used with caution and content should be monitored. I would not hesitate to report someone if I thought they were breaching confidentiality/data protection (Participant 111).

Some participants are more optimistic regarding the professional use of social media:

As long as it is used professionally and under NMC standards adhering to confidentiality, the benefits can be endless. (Participant 23).

I love it! I have met so many great like‐minded healthcare professionals and been given so many amazing opportunities to help develop my career that never would have occurred if I didn't post on social media. (Participant 258).

Others are more cautious and put some strategies in place to use social media. Having a professional account and handle with a name can help:

I use Twitter with my real name and photo as a professional/networking platform – whereas Instagram I use only for personal reasons and don't post anything identifying me (no photo or real name). I have found Twitter really good for sharing resources, networking and exchanging interesting views – but it can get a bit too heated/stressful when people attack each other if they don't agree. (Participant 76).

It is also important to look at account settings.

I have social media accounts but never post on them because I'm cautious of my privacy and anything I put online if my account is public could be accessed by anyone. I also think it's really easy to accidentally reveal too much on social media. (Participant 124).

Moderating your post is considered important.

I enjoy browsing social media in my spare time but rarely post due to the impacted a wrong decision can affect your future (Participant 267).

Maintaining a level of privacy is another strategy used:

I prefer not to mention I am a nursing student on Social Media, however I am part of a closed nursing group forum on Facebook. Other than that I do not post anything nursing related (Participant 287).

I have however changed my name on all social media accounts so that it is different from my name badge in work so that no patients or relatives can find me if they were to look for me. (Participant 325).

## 6 DISCUSSION

This study aimed to identify and report the use of social media among pre‐registration (pre‐licence) UK student nurses and aligned it to the social media diffusion timeline by using Rogers' (2003) diffusion of innovation theory as a theoretical framework. Social identity theory was also used to analyse students' experience within the diffusion of innovation theory. Four themes emerged: (1) Communication, (2) Selfcare, (3) Learning, (4) Professionalism. The discussion was structured following the diffusion of innovation theoretical framework (Rogers, 2003). Rogers' diffusion process theory contains four elements (Rogers, 2003):

1. The innovation. Rogers defined innovation as: “an idea, practice, or object that is perceived as new by an individual or other unit of adoption” (2003, p. 12). Social media in pre‐registration nursing students is the innovation explored in this study.
2. The innovation's communication channels. Ratheeswari (2018) defines communication as how users share information to network and learn. Communication channels are directly influencing the innovation spread among users and comprise different ways of communication. Tarde (1903) first identified the concepts of homophily and heterophily which impact communication channels. Lazarsfeld and Merton (1954) developed those concepts further. Homophily is people's affinity or interaction with people like themselves, whereas heterophily is people's affinity or interaction with people different to themselves (Lazarsfeld & Merton, 1954).
3. Time. Time is an important factor in understanding the diffusion of innovation and the rate of adoption. Rogers classified users into five categories: (1) Innovator, (2) Early adopters, (3) Early majority, (4) Late majority, (5) Laggards. Those categories refer to how early or late users are adopting an innovation.
4. Social system. The social system is probably the most intricate element of social media diffusion of innovation. Due to the specificity of nursing education, additional factors influence student nurses' use of social media experience. From the 4 themes that emerged, three social systems are present in UK student nurses' social media experience: personal social system, student social system and professional social system.

### 6.1 Social media and communication

The adoption rate of innovation is influenced by social media perceived attributes. Rogers (2003) identified five perceived attributes: (1) Relative advantages, (2) Compatibility, (3) Complexity, (4) Trialability, (5) Observability. On these attributes, social media are attractive. Social media present clear advantages to student nurses worldwide, as it allows communication and information sharing with friends, family, peers and colleagues independently of location (O'Connor et al., 2022; Vizcaya‐Moreno & Pérez‐Cañaveras, 2020; Zachos et al., 2018). Alharbi et al. (2021) integrative review included 12 papers and found that the most common reason for student nurses using social media was its ability to keep them connected and in touch with others.

Social media are highly compatible, the only requirement to use social media is a smart device (phone, tablet, laptop, computer) and internet access (which we acknowledge can be a barrier where digital poverty exists). Using social media is easy and requires minimum training, which makes them accessible to a wide range of people. They are free to access and widely accessible on the network to be trialled. Due to the nature of social media, they are highly observable and part of the daily life of most of the younger generation of nursing students.

In social media innovation and from the data, we can identify one homophily group. Students who have the same objectives and use social media to achieve their aims. The means of communication and the similarities between members of a homophily group increase communication efficiency. Participants in this study like those in Aleksandrova and Parusheva (2019) use instant messaging systems, and interpersonal channels to share the perceived attributes of social media. The findings demonstrate the use of social media for communication exists and its diffusion is advancing. Those channels are fast, and the trust between students/friends regarding their experience and information is more likely to add weight to accepting the information as true.

Social media use and presence in student nurses' communication have increased. It is more than a platform for social relationships, it is an information hub transmitting and relaying information on news, health and topical issues (Hashim et al., 2020) Student nurses have been innovators and early adopters and use social media increasingly in their communication.

In terms of social system, student nurses mostly use social media for communication with family and friends and to keep themselves informed within their personal social system. In this system, social media are regulated by societal rules which can fluctuate with the generations and what is acceptable can differ within the national and international laws and regulations of social media.

### 6.2 Social media and self‐care

Social media are well‐known for communication and keeping people connected. This study identified that it also impacts student nurses' self‐care. A similar phenomenon was identified in a Caribbean student nurses' population (Cathala et al., 2022). Both findings seem to indicate that social media is now beyond straightforward communication and student nurses are creating not only social communities but communities of practice and professional support groups using social media as a coping mechanism in challenging situations. These communities can also give student nurses a sense of belonging to the nursing profession.

As seen in the social media and communication theme, there are many positive attributes that social media provides to students. They can strengthen communication, look after each other and increase support. Student nurses enjoy social media attributes to boost their self‐esteem, morale, support and fight against loneliness. Social media enables students to access those positive attributes for free, which is important to students with the current constantly increasing cost of living and the stressful and challenging student nurses' experiences. Despite social media sometimes being presented as negative, unhealthy and potentially hazardous (Kross et al., 2021), this study identified how student nurses found support for their well‐being through social media engagement. Worthy of note is that Appel et al. (2020) meta‐analysis evidence does not support those negative claims. Social media and self‐care impact all three social systems and support students in their personal, student and professional social systems.

### 6.3 Social media and learning

Social media were first created in 1997, and 10 years later in 2008, Skiba published the first article demonstrating an interest in the use of social media in nursing education with an increased interest since (Chugh et al., 2021). Existing literature and our data demonstrate that students already use social media for learning and could be seen as innovators or early adopters (Ansari & Khan, 2020; Sutherland et al., 2020). However, students and universities are not at the same level of adoption of social media and universities are laggards in using social media for learning. Some universities advise students to not use social media to prevent any challenges without mentioning the potential positive attributes that social media could provide to students. A scoping review from Chugh et al. (2021) shows that there is institutional and cultural resistance as well as a limited understanding of pedagogical attributes of social media and recommends an increase in support and training for academic staff.

This disconnect between students and universities in the use of social media could be explained by the heterogeneity of the 2 groups and how differently they are operating. Students' adoption of innovation is faster than university as peer students' positive experience suffice for them to use social media. Compared to universities that have a much slower adoption due to their research and quality assurance processes. The population composition and specifically the generations could also impact the adoption rate in both populations.

Social media and learning impact all three social systems, with a greater impact on their student and professional system while being student nurses.

### 6.4 Social media and professionalism

Social media is rapidly diffused in the professional sphere, used professionally in multiple areas and is nowadays a requirement for businesses (Dwivedi et al., 2023). Nursing does not escape this phenomenon. However, from a safety and regulatory perspective, the professional misuse of social media and its negative attributes were focused on addressing misbehaviours. Those negative events triggered national guidance from key UK stakeholders including regulators (NMC, 2023), colleges (RCN, 2020), societies, hospitals and universities. Internationally guidance is also available for example from the Australian (Nursing and Midwifery Board of Australia, 2019) and American (National Council of States Board of Nursing, 2018). Unfortunately, these guidelines often lack depth and clarity and are not supported by teaching or training programmes at the University level (Cathala et al., 2022; Marsh, 2023). However, social media are widely and successfully used for health campaigns, marketing and research. This reinforces the dichotomy of social media “use it but not use it” rather than supporting and teaching student nurses to acquire professional proficiencies on social media. Unfortunately, the positive attributes and potential benefits of social media were less investigated. Becoming a nurse and being a registered professional adds complexity, student nurses and nurses must adhere to the regulatory body regulations, requirements and Code (NMC, 2018). However, this Code does not apply to their professional system only but to their personal, student and professional systems. Our participants clearly expressed the anxiety that social media from any social system could impact others. Interestingly, our participants strongly commented on these challenges, which were not identified in a similar study with Caribbean student nurses undertaken by Cathala et al. (2022). This difference could suggest a different diffusion of innovation and therefore different support training and guidance. Further development of student nurses' training and support in developing proficiencies in using social media to acquire a complete nurse identity is required.

### 6.5 Social system

**FIGURE 2:**
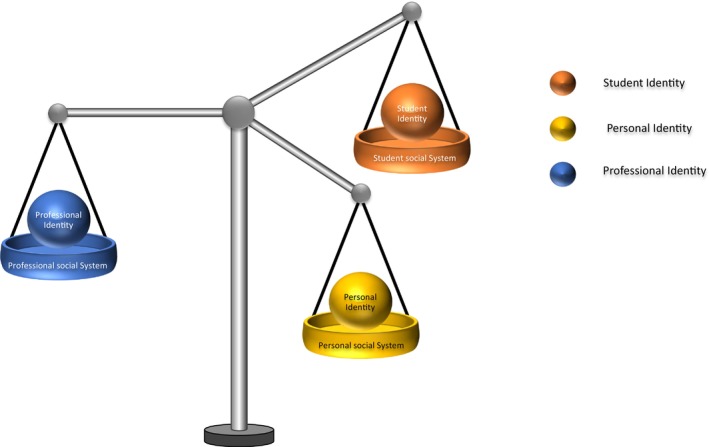
UK student nurses' social identity.

From a social identity theory perspective, it appears that the Social Identity of each social system differs, making the student nurse's Social Identity challenging. The Personal Identity does not fit the Professional Identity, and the Student Identity seems to expect more than the Professional Identity. O'Connor et al. (2022) stressed the digital professionalism challenges that student nurses are facing; these are also described by Wang et al. (2019) for registered nurses internationally. More than identifying the challenges faced by student nurses, this study offers an understanding of the reason for those challenges. There is a disconnect between the different identities and social systems that could explain the challenges that some student nurses navigate. This contrasts with the regulatory body regulations and guidelines, for example UK's Nursing and Midwifery Council (NMC) Code (2018) which should apply across the systems without differentiation. Any misbehaviour related to any social system can impact other systems and identities, creating an imbalance in UK student nurses' Social Identity (Figure 2). One of the biggest concerns that UK student nurses report is the negative impact social media can have on their careers and could impact social media diffusion. Support and guidance in developing UK student nurses' Social Identity in the different online social systems could support and further develop their online identity, self‐care, learning and professionalism, improving their experience of social media and developing future nurses' professionalism in an increasingly connected world. Poor social identity on social media could result not only in lacking professionalism but also in missing benefits such as peer/colleague support, and a sense of belonging and could experience some disconnect with the online community and younger professionals. However, appropriate demonstration of social identity for each social system could positively affect and support the formation, development, maintenance or change of student nurses' Social Identity. Appropriate social identity adoption on social media could support student education, professionalism and access to the nursing community and act as a catalyser of their professional career.

Cathala et al. (2022) examined Caribbean student nurses' social participation using social media and reported similar findings, (self‐care, communication and learning) except for social media and professionalism. Our UK sample compared to their Caribbean counterparts appeared to have more concerns about adopting social media and its impact on their social identity and specifically their professional identity. This could demonstrate that the diffusion process can be influenced by specific characteristics, context, politics and culture, which suggests further study is required to understand the complexity of social media diffusion.

This new insight in social media diffusion should be used to inform nursing education curricula, communication policies and training by integrating social media into curriculum delivery to develop social identity understanding and skills and also the development of training, support, guidelines and policies.

## 7 LIMITATIONS

The authors recognize limitations in this study. It investigates UK pre‐registration student nurses only and not registered nurses or registered nurses in education. A purposive sample was used. Diffusion of innovation theory measures the spread of innovation over time, and it can be considered that we did not measure it over time; however, we used it as a theoretical framework as the foundation of our reflexive thematic approach. The literature available allowed us to identify the diffusion of social media use from its creation to the time of data collection. This knowledge of its diffusion has been instrumental in the reflexive process and the discovery of the themes presented in this paper. We acknowledge that the new knowledge presented in the paper is a snapshot of the diffusion of social media timeline. However, this is still part of the timeline of its diffusion and where students were at the time of the data collection. This adds to the understanding of the diffusion of social media and brings new perspectives on the support and training that students require.

The use of diffusion of innovation theory has some limitations such as pro‐innovation bias which imply that the innovation should be adopted by all members of the social system. Recognition that social media will not be suitable for all members and its use must remain within the Code (NMC, 2018). Time is an important factor in innovation diffusion in terms of recalling information and data but also limiting the possible finding if the adoption of diffusion is not completed.

## 8 CONCLUSION

The diffusion of social media among UK student nurses is well on the way in different aspects of UK student nurses' lives (communication, learning and self‐care). However, the diffusion seems to slow down when it comes closer to the professional system. UK student nurses face some challenges in developing their social identity to fully enjoy social media attributes in the different systems. They clearly express concerns that one social system can negatively impact another and have repercussions on a personal and/or professional level and therefore prevent UK student nurses from developing social media proficiencies. Guidance and support should be offered to UK student nurses to develop their social identity across the different systems and this diffusion can help to educate student nurses and future professionals in a globally connected world.

## FUNDING INFORMATION

This research received no specific grant from any funding agency in the public, commercial, or not‐for‐profit sectors.

## CONFLICT OF INTEREST STATEMENT

No conflict of interest.

### PEER REVIEW

The peer review history for this article is available at https://www.webofscience.com/api/gateway/wos/peer‐review/10.1111/jan.16405.

## Data Availability

The data that support the findings of this study are available on request from the corresponding author. The data are not publicly available due to privacy or ethical restrictions.

## References

[jan16405-bib-0001] Aleksandrova, Y. G. , & Parusheva, S. S. (2019). Social media usage patterns in higher education institutions–an empirical study. International Journal of Emerging Technologies in Learning (Online), 14(5), 108.

[jan16405-bib-0002] Alharbi, M. , Kuhn, L. , & Morphet, J. (2021). Nursing students' engagement with social media as an extracurricular activity: An integrative review. Journal of Clinical Nursing, 30(1–2), 44–55. 32956547 10.1111/jocn.15503

[jan16405-bib-0003] Ansari, J. A. N. , & Khan, N. A. (2020). Exploring the role of social media in collaborative learning the new domain of learning. Smart Learning Environments, 7(1), 9.

[jan16405-bib-0004] Appel, M. , Marker, C. , & Gnambs, T. (2020). Are social media ruining our lives? A review of meta‐analytic evidence. Review of General Psychology, 24(1), 60–74.

[jan16405-bib-0005] Bailey, J. (2008). First steps in qualitative data analysis: Transcribing. Family Practice, 25, 127–131. 18304975 10.1093/fampra/cmn003

[jan16405-bib-0007] Braun, V. , & Clarke, V. (2006). Using thematic analysis in psychology. Qualitative Research in Psychology, 3(2), 77–101. 10.1191/1478088706qp063oa

[jan16405-bib-0008] Braun, V. , & Clarke, V. (2012). Thematic analysis, APA handbook of research methods in psychology. In H. Cooper , P. M. Camic , D. L. Long , A. T. Panter , D. Rindskopf , & K. J. Sher (Eds.), Research designs: Quantitative, qualitative, neuropsychological, and biological (Vol. 2, pp. 57–71). American Psychological Association. 10.1037/13620-004

[jan16405-bib-0009] Braun, V. , & Clarke, V. (2022). Conceptual and design thinking for thematic analysis. Qualitative Psychology, 9(1), 3–26. 10.1037/qup0000196

[jan16405-bib-0010] Cathala, X. , & Moorley, C. (2023). Diffusion of social media in nursing education: A scoping review. Nurse Education Today, 127, 105846. 10.1016/j.nedt.2023.105846 37236015

[jan16405-bib-0011] Cathala, X. , Ocho, O. N. , Mcintosh, N. , Watts, P. N. , & Moorley, C. (2022). An exploration of social participation in Caribbean student nurses' use of social media in their learning journey. Journal of Advanced Nursing, 79(8), 2900–2910. 36401572 10.1111/jan.15499

[jan16405-bib-0012] Cathala, X. , Ocho, O. N. , Moorley, C. , & Watts, P. N. (2021). Demographic profiling of Caribbean and United Kingdom student nurses' use of social media for professional development. Journal of Professional Nursing, 37(6), 1036–1043. 34887020 10.1016/j.profnurs.2021.08.013

[jan16405-bib-0013] Cathala, X. , Ocho, O. N. , Watts, P. N. , & Moorley, C. (2021). International student nurses' use of social media for learning: A cross sectional survey. Nurse Education Today, 107, 105160. 34607295 10.1016/j.nedt.2021.105160

[jan16405-bib-0015] Chugh, R. , Grose, R. , & Macht, S. A. (2021). Social media usage by higher education academics: A scoping review of the literature. Education and Information Technologies, 26(1), 983–999.

[jan16405-bib-0016] Daigle, A. (2020). Social media and professional boundaries in undergraduate nursing students. Journal of Professional Nursing, 36(2), 20–23. 10.1016/j.profnurs.2019.08.007 32204855

[jan16405-bib-0017] Dwivedi, Y. K. , Ismagilova, E. , Rana, N. P. , & Raman, R. (2023). Social media adoption, usage and impact in business‐to‐business (B2B) context: A state‐of‐the‐art literature review. Information Systems Frontiers, 25, 1–23.

[jan16405-bib-0018] Greenhow, C. , & Galvin, S. (2020). Teaching with social media: Evidence‐based strategies for making remote higher education less remote. Information and Learning Science, 121(7/8), 513–524.

[jan16405-bib-0019] Hampton, D. , Welsh, D. , & Wiggins, A. T. (2020). Learning preferences and engagement level of generation Z nursing students. Nurse Educator, 45(3), 160–164. 31219957 10.1097/NNE.0000000000000710

[jan16405-bib-0020] Hashim, S. , Masek, A. , Abdullah, N. S. , Paimin, A. N. , & Muda, W. H. N. W. (2020). Students' intention to share information via social media: A case study of COVID‐19 pandemic. Indonesian Journal of Science and Technology, 5(2), 236–245.

[jan16405-bib-0021] International Council of Nurses . (2015). Position Statement Nurses and Social Media. https://www.icn.ch/sites/default/files/2023‐04/E10a_Nurses_Social_Media.pdf

[jan16405-bib-0023] Kross, E. , Verduyn, P. , Sheppes, G. , Costello, C. K. , Jonides, J. , & Ybarra, O. (2021). Social media and well‐being: Pitfalls, progress, and next steps. Trends in Cognitive Sciences, 25(1), 55–66. 33187873 10.1016/j.tics.2020.10.005

[jan16405-bib-0024] Lazarsfeld, P. F. , & Merton, R. K. (1954). Friendship as a social process: A substantive and methodological analysis. Freedom and Control in Modern Society, 18(1), 18–66.

[jan16405-bib-0025] Lefebvre, C. , McKinney, K. , Glass, C. , Cline, D. , Franasiak, R. , Husain, I. , Pariyadath, M. , Roberson, A. , McLean, A. , & Stopyra, J. (2020). Social media usage among nurses: Perceptions and practices. The Journal of Nursing Administration, 50(3), 135–141. 10.1097/NNA.0000000000000857 32049701

[jan16405-bib-0026] Lincoln, Y.S. and Guba, E.G. , (1985). *Naturalistic inquiry*. Sage.

[jan16405-bib-0028] Maitri, W. S. , Suherlan, S. , Prakosos, R. D. Y. , Subagja, A. D. , & Ausat, A. M. A. (2023). Recent trends in social media marketing strategy. Jurnal Minfo Polgan, 12(1), 842–850.

[jan16405-bib-0029] Marsh, A. (2023). Social me dia use by midwives–an untapped potential? Doctoral dissertation, Bournemouth University.

[jan16405-bib-0030] Mohr, E. S. , & Mohr, K. A. (2018). The ABCs of the XYZs: Adding a critical dimension to contemporary teacher education. Journal of Education and Training Studies, 5, 182–189.

[jan16405-bib-0031] Moorley, C. , & Cathala, X. (2019). How to appraise qualitative research. Evidence‐Based Nursing, 22(1), 10–13. 30504448 10.1136/ebnurs-2018-103044

[jan16405-bib-0032] Mude, G. , & Undale, S. (2023). Social media usage: A comparison between generation Y and generation Z in India. International Journal of E‐Business Research (IJEBR), 19(1), 1–20.

[jan16405-bib-0033] National Council of States Board of Nursing . (2018). A Nurse's Guide to the Use of Social Media. https://www.ncsbn.org/brochures‐and‐posters/nurses‐guide‐to‐the‐use‐of‐social‐media

[jan16405-bib-0034] Nursing and Midwifery Board of Australia . (2019). Social media: How to meet your obligations under the National law. https://www.nursingmidwiferyboard.gov.au/Codes‐Guidelines‐Statements/Codes‐Guidelines/Social‐media‐guidance.aspx

[jan16405-bib-0035] Nursing and Midwifery Council . (2018). The code. https://www.nmc.org.uk/standards/code/

[jan16405-bib-0036] Nursing and Midwifery Council . (2023). Guidance on using social media responsibly. https://www.nmc.org.uk/standards/guidance/social‐media‐guidance/

[jan16405-bib-0037] O'Connor, S. , Odewusi, T. , Smith, P. M. , & Booth, R. G. (2022). Digital professionalism on social media: The opinions of undergraduate nursing students. Nurse Education Today, 111, 105322. 10.1016/j.nedt.2022.105322 35263709

[jan16405-bib-0038] Ohara, M. R. (2023). The role of social media in educational communication management. Journal of Contemporary Administration and Management (ADMAN), 1(2), 70–76. 10.61100/adman.v1i2.25

[jan16405-bib-0039] Payne, G. , & Payne, J. (2011). Key concepts in social research. Sage.

[jan16405-bib-0040] Picton, R.B. (2023), Mind The Gap. https://openresearch.lsbu.ac.uk/download/20b52120200e01ca049711cdf627b00d5e2e11222e1dbb890b20f000321ad692/2718430/MindtheGapThesissubmission15.09.23v2.pdf

[jan16405-bib-0041] Price, A. M. , Devis, K. , LeMoine, G. , Crouch, S. , South, N. , & Hossain, R. (2018). First year nursing students use of social media within education: Results of a survey. Nurse Education Today, 61, 70–76. 29179050 10.1016/j.nedt.2017.10.013

[jan16405-bib-0042] Ratheeswari, K. (2018a). Information communication technology in education. Journal of Applied and Advanced Research, 3(1), 45–47.

[jan16405-bib-0043] Rogers, E. M. (2003). Diffusion of innovations (Fifth ed.). Free Press, A Division of Simon & Schuster, Inc.

[jan16405-bib-0044] Royal College of Nursing . (2020). Social Media Use. https://www.google.com/url?sa=t&rct=j&q=&esrc=s&source=web&cd=&ved=2ahUKEwjIvKCHgJeGAxUbQUEAHb4RCfwQFnoECC4QAQ&url=https%3A%2F%2Fwww.rcn.org.uk%2F‐%2Fmedia%2Froyal‐college‐of‐nursing%2Fdocuments%2Fcountries‐and‐regions%2Fwales%2F2020%2Fjune%2Fsocial‐media‐use‐guidance‐jun‐2020.pdf%3Fla%3Den&usg=AOvVaw0DoRc4sAChs5L9BpNnILzr&opi=89978449

[jan16405-bib-0045] Ryan, G. (2016). International perspectives on social media guidance for nurses: A content analysis. Nursing Management, 23(8), 28–35. 27905234 10.7748/nm.2016.e1555

[jan16405-bib-0046] Scott, N. , & Goode, D. (2020). The use of social media (some) as a learning tool in healthcare education: An integrative review of the literature. Nurse Education Today, 87, 104357. 10.1016/j.nedt.2020.104357 32032837

[jan16405-bib-0047] Steers, M.L.N. and Gallups, S.F. , 2020. Ethical tipping point: Nurses' presence on social media. Nursing *2023*, 50(12), pp.52–54. 10.1097/01.NURSE.0000694768.02007.f1 33497095

[jan16405-bib-0048] Sutherland, K. , Terton, U. , Davis, C. , Driver, C. , & Visser, I. (2020). Academic perspectives and approaches to social media use in higher education: A pilot study. International Journal of Teaching and Learning in Higher Education, 32(1), 1–12.

[jan16405-bib-0049] Tajfel, H. , & Turner, J. C. (2004). The social identity theory of intergroup behavior. In Political psychology (pp. 276–293). Psychology Press.

[jan16405-bib-0050] Tarde, G. (1903). The Laws of imitation. Translated by Elsie Clews.

[jan16405-bib-0051] Terzi̇, B. , Bulut, S. , & Kaya, N. (2019). Factors affecting nursing and midwifery students' attitudes toward social media. Nurse Education in Practice, 35, 141–149. 10.1016/j.nepr.2019.02.012 30825708

[jan16405-bib-0052] Vizcaya‐Moreno, M. F. , & Pérez‐Cañaveras, R. M. (2020). Social media used and teaching methods preferred by generation z students in the nursing clinical learning environment: A cross‐sectional research study. International Journal of Environmental Research and Public Health, 17(21), 8267. 33182337 10.3390/ijerph17218267 PMC7664855

[jan16405-bib-0053] Wang, Z. , Wang, S. , Zhang, Y. , & Jiang, X. (2019). Social media usage and online professionalism among registered nurses: A cross‐sectional survey. International Journal of Nursing Studies, 98, 19–26. 31255853 10.1016/j.ijnurstu.2019.06.001

[jan16405-bib-0054] Zachos, G. , Paraskevopoulou‐Kollia, E. A. , & Anagnostopoulos, I. (2018). Social media use in higher education: A review. Education in Science, 8(4), 194.

[jan16405-bib-0055] Zhu, X. , Hu, H. , Xiong, Z. , Zheng, T. , Li, L. , Zhang, L. , & Yang, F. (2021). Utilization and professionalism toward social media among undergraduate nursing students. Nursing Ethics, 28(2), 297–310. 10.1177/0969733020952105 32975494

